# Supplementary material for: Atherosclerotic cardiovascular disease thresholds for statin initiation among people living with HIV in Thailand: A cost-effectiveness analysis
Source: PLoS One. 2021 Sep 9;16(9):e0256926. doi: 10.1371/journal.pone.0256926 (PMC8428548; doi:10.1371/journal.pone.0256926)
Supplement: S1 File — (DOCX) [file pone.0256926.s001.docx]

**Atherosclerotic cardiovascular disease thresholds for statin initiation among people living with HIV in Thailand: A cost-effectiveness analysis**

- **Supplementary Material**

**Model Summary**

We used individual patient data from all Thai sites contributing to the TREAT Asia HIV Observational Database (TAHOD), the reduced Data-collection on Adverse Effects of Anti-HIV Drugs (D:A:D) cardiovascular disease risk equation, and published literature to estimate medical costs and quality-adjusted life-years (QALYs) among people living with HIV (PLHIV) in Thailand.

**Study Population**

TAHOD is an ongoing collaboration of 21 HIV clinics in the Asia-Pacific region that is part of the International Epidemiology Databases to Evaluate AIDS Asia-Pacific.[1] Participating clinics follow local guidelines and regulations regarding patient consent and ethics review. Our study population included patients enrolled in TAHOD at one of the four Thai sites involved (Ramathibodi Hospital, Bangkok; HIV-NAT Research Collaboration/Thai Red Cross AIDS Research Centre, Bangkok; Research Institute for Health Sciences, Chiang Mai; and Chiangrai Prachanukroh Hospital, Chiang Rai) who had documentation of at least one clinic visit between 1 January 2013 and 1 September 2019 and who, at their last documented clinic visit, were aged 35-75 years, had no history of ASCVD, had been using antiretroviral therapy (ART) for at least 6 months, and had a CD4 cell count >100 cells/mm^3^. Stable ART was included as a selection criterion as this should be prioritized by PLHIV over atherosclerotic cardiovascular disease (ASCVD) risk management. Individuals with missing ASCVD risk factor data were excluded. For PLHIV in the study population already using a statin, their cholesterol levels were “de-statinized” based on the mean statin efficacy reported by the Cholesterol Treatment Trialists Collaboration.[2] Supplementary Table 1 further characterizes our study population.

**Supplementary Table 1 – Study population characteristics at beginning of simulation**

| **Characteristic** | **N=1,379** | |
| --- | --- | --- |
| Sex | Male | 700 (50.8) |
| Age, years | Median (IQR) | 48.4 (43.2, 54.7) |
| Mode of HIV exposure | Heterosexual | 1,222 (88.6) |
|  | Homosexual | 118 (8.6) |
|  | Intravenous drug use | 16 (1.2) |
|  | Other | 23 (1.7) |
| Family history of ASCVD | Yes | 106 (7.7) |
| Diabetic | Yes | 102 (7.4) |
| Chronic kidney disease | Yes | 142 (10.3) |
| Current smoker | Yes | 196 (14.2) |
| Ever smoked | Yes | 484 (35.1) |
| Systolic blood pressure, mmHg | Median (IQR) | 127 (116, 137) |
| Using antihypertensive medication | Yes | 216 (15.7) |
| Total cholesterol, mg/dL | Median (IQR) | 201 (174, 232) |
| LDL-C, mg/dL | Median (IQR) | 135 (108, 162) |
| HDL-C, mg/dL | Median (IQR) | 50 (39, 62) |
| CD4 cell count, cells/mm^3^ | Median (IQR) | 573 (427, 745) |
| Antiretroviral therapy regimen | NNRTI-based | 1,164 (84.4) |
|  | PI-based | 191 (13.9) |
|  | Other | 24 (1.7) |
| D:A:D risk score, 10-year risk of CVD^a^ | <5% | 835 (60.6) |
|  | ≥5 – 7.49% | 211 (15.3) |
|  | ≥7.5% - 9.99% | 113 (8.2) |
|  | ≥10% | 220 (16.0) |

All values are No. (%) unless otherwise specified. ^a^Published D:A:D 5-year risk equation converted to 10-year risk equation by replacing the absolute 5-year survival risk (0.9853) with the absolute 10-year survival risk (0.9697).[3] IQR, interquartile range; ASCVD, atherosclerotic cardiovascular disease; LDL-C, low density lipoprotein cholesterol; HDL-C, high density lipoprotein cholesterol; NNRTI, non-nucleoside reverse transcriptase inhibitor; PI, protease inhibitor; D:A:D, Data-collection on Adverse Effects of Anti-HIV Drugs

**Model Structure and Model Parameters**

To inform the structure of our model and select appropriate parameter estimates we searched PubMed, applicable guideline documents, and abstracts presented at appropriate international conferences. Material in English published or presented after 1999 was considered potentially relevant. We conducted two distinct PubMed searches. The first term used was (“cardiovascular” or “heart” or “coronary” or “cerebrovascular” or “myocardial” or “stroke”) AND (“HIV” or “human immunodeficiency virus”) and the second term was (“statin” or “pravastatin” or “pitavastatin” or “atorvastatin” or "simvastatin" or “fluvastatin” or “lovastatin” or “rosuvastatin”) AND (“cost-effectiveness” or “cost-utility” or “cost-benefit” or “cost”). The highest level of evidence available was used to parameterize the model. Where multiple divergent estimates were available, we adopted a wide sensitivity range to include all estimates.

We developed a discrete-state microsimulation model that randomly selected (with replacement) 100,000 PLHIV from our TAHOD study population and simulated their experience over time. The model assumed the Thai healthcare sector perspective and applied a lifetime horizon (with half-cycle correction) to allow sufficient event accumulation to compare treatment strategies. The model structure is depicted in Supplementary Figure 1.

Key model parameters are described in Table 1 of the main text. Primary ASCVD risk was calculated using the reduced D:A:D CVD risk equation.[4] This is the only HIV-specific risk equation recommended by the American College of Cardiology/American Heart Association for PLHIV.[5] We used the reduced D:A:D equation (which is based on age, sex, diabetes status, family history of ASCVD, current smoking status, past smoking status, total cholesterol, high density lipoprotein cholesterol, systolic blood pressure, and CD4 count) rather than the full equation (which also includes ART) because the reduced model is recommended for PLHIV exposed to ART for more than 5 years.[4] We modelled the effectiveness of statins through the simulated change in low density lipoprotein cholesterol (LDL-C). We assumed fully adherent individuals using high-intensity and moderate-intensity statins would achieve LDL-C reductions of 54.8% and 44.0%, respectively.[2, 6] Statin adherence was assumed to be 86.7% in the first year of statin use, 72.6% in the second, 61.1% in the third, 56.6% in the fourth, and 58.1% in all years thereafter.[7] The mg/dL reduction in LDL-C was converted to a percentage of 38.6mg/dL and the ASCVD risk reduction associated with 38.6mg/dL LDL-C reduction was multiplied by this percentage to ascertain each individuals ASCVD risk reduction. The reduction in ASCVD risk associated with LDL-C reduction varied by individuals 5-year CVD risk (based on the published D:A:D equation) and the ASCVD event type being evaluated (Supplementary Table 2).[2] We assumed PLHIV would only incur the cost of statins, exhibit side effects of statins, and benefit from statin LDL-C cholesterol reduction they were using a statin and hence these parameters were adjusted in line with the decline in adherence over time.

With each annual cycle we added one year of age, and assumed age- and sex-specific changes in systolic blood pressure [8] and rates of diabetes,[9] and age-specific rates of smoking cessation.[10] All other variables used to calculate ASCVD risk were kept constant over time. Since the risk score defines CVD as a composite of coronary intervention, T1MI, stroke (ischemic or hemorrhagic) or other cardiovascular death, we apportioned the calculated risk into individual event types based on the proportions reported in Friis-Moller *et al*.[4] Background mortality rates were based on those of the Asian HIV population (Supplementary table 3).[11]

Recurrent event probabilities were based on reported event rates in the years after an incident event and were primarily based on published estimates for the general population.[12-21] Further details are available in our earlier publication.[22] We did not use the D:A:D score or an HIV-specific hazard ratio as current evidence suggests that risk factors for primary ASCVD differ substantially from those of recurrent ASCVD.[18, 23-25] Statins were assumed to be used as secondary ASCVD prevention in both our control and intervention arms.

Since statins have been associated with an increased risk of hemorrhagic stroke,[2] diabetes [26] and myopathy,[27] we assumed hazard ratios and costs for these adverse events consistent with literature from the general population (see Table 1 in main text).

Individuals accumulated costs and benefits up until their death, and future costs and benefits were discounted at 3% per year.

**Supplementary Figure 1 – Core model structure**

**
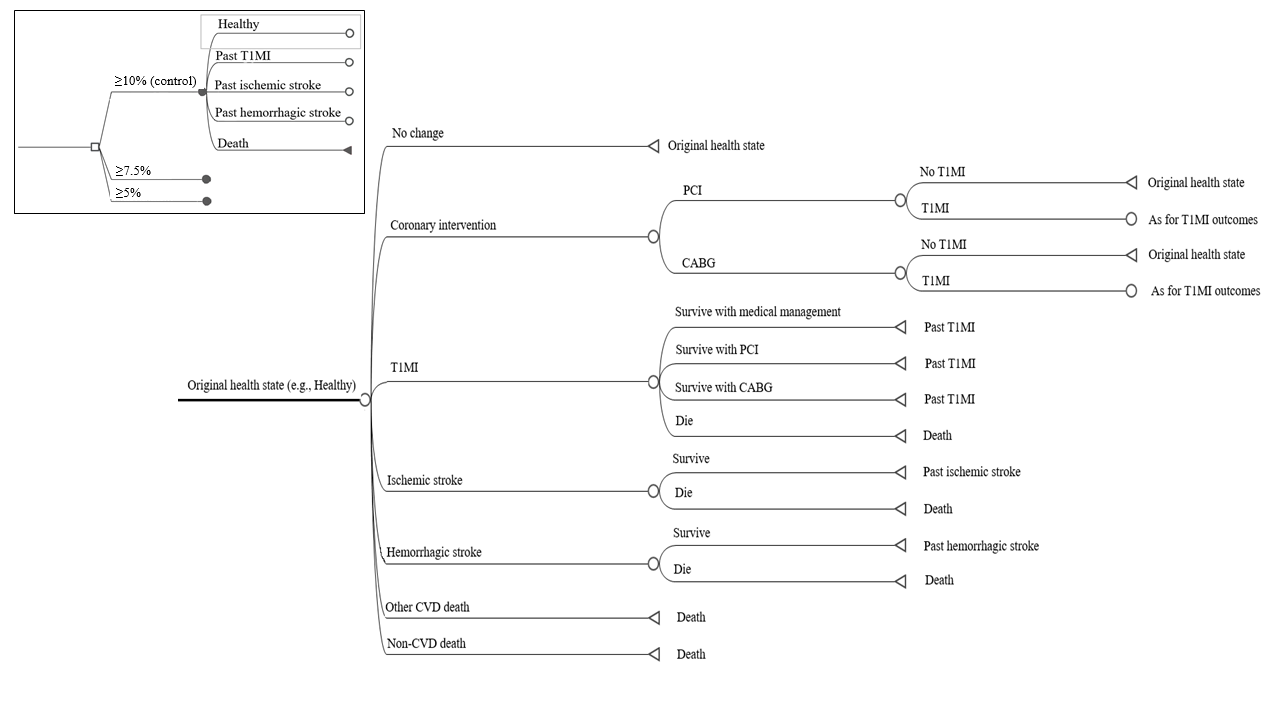
**

The box in the top left corner shows the model comprised three intervention arms branching from a decision node (unfilled square): ASCVD risk threshold for statin initiation ≥10% (control group), ≥7.5% and ≥5%. The Markov node (filled circle) for each intervention arm included five chronic states: healthy, past T1MI, past ischemic stroke, past hemorrhagic stroke, or death (absorbing state; filled triangle). For simplicity, the chronic states are only depicted in the sub-tree for the no statin arm. In the healthy state (highlighted by a grey rectangle in the top left box and expanded in the main tree), individuals transitioned in annual cycles to an acute state of no change, coronary intervention, T1MI, ischemic stroke, hemorrhagic stroke, other CVD death, or non-CVD death. The acute states branching from past T1MI, past ischemic stroke, and past hemorrhagic stroke are the same as those of the healthy state but without coronary intervention as an option. The unfilled circles represent chance nodes where each branch to the right of the node is assigned a pre-specified probability. The unfilled triangles represent terminal nodes and indicate where an individual ends up at the conclusion of an annual cycle and the state in which they begin the next annual cycle. Individuals accumulated costs and benefits up until their death or the time horizon, whichever came first. T1MI, type 1 myocardial infarction; ASCVD, atherosclerotic cardiovascular disease; PCI, percutaneous coronary intervention; CABG, coronary artery bypass graft.

**Supplementary Table 2 – Reduction in ASCVD probability with 38.6mg/dL LDL-C reduction**

| **5-year CVD risk^a^** | **Intervention** | **T1MI** | **Ischemic stroke** | **CVD death** |
| --- | --- | --- | --- | --- |
| <5% | 0.48 | 0.43 | 0.21 | 0.38 |
| 5% to <10% | 0.37 | 0.39 | 0.21 | 0.31 |
| 10% to <20% | 0.25 | 0.23 | 0.21 | 0.21 |
| 20% to <30% | 0.21 | 0.23 | 0.21 | 0.19 |
| ≥30% | 0.24 | 0.22 | 0.21 | 0.21 |

^a^ Based on the 5-year D:A:D equation published by Friis-Moller *et al*.[4] ASCVD, atherosclerotic cardiovascular disease; LDL-C, low density lipoprotein cholesterol; T1MI, type 1 myocardial infarction

**Supplementary Table 3 – Background annual mortality probability**

| **Age** | **Men CD4 100-199** | **Men CD4 200-249** | **Men CD4 250-349** | **Men CD4 350-500** | **Men CD4 >500** | **Women CD4 100-199** | **Women CD4 200-249** | **Women CD4 250-349** | **Women CD4 350-500** | **Women CD4 >500** |
| --- | --- | --- | --- | --- | --- | --- | --- | --- | --- | --- |
| 30 | 0.016 | 0.011 | 0.007 | 0.009 | 0.005 | 0.008 | 0.006 | 0.004 | 0.005 | 0.003 |
| 40 | 0.018 | 0.012 | 0.008 | 0.010 | 0.006 | 0.009 | 0.006 | 0.004 | 0.005 | 0.003 |
| 50 | 0.028 | 0.019 | 0.012 | 0.016 | 0.009 | 0.014 | 0.010 | 0.006 | 0.008 | 0.004 |
| 60 | 0.080 | 0.061 | 0.046 | 0.056 | 0.039 | 0.052 | 0.039 | 0.029 | 0.036 | 0.025 |
| 70 | 0.142 | 0.115 | 0.090 | 0.108 | 0.080 | 0.102 | 0.081 | 0.063 | 0.076 | 0.056 |
| 80 | 0.252 | 0.215 | 0.179 | 0.206 | 0.166 | 0.197 | 0.165 | 0.137 | 0.160 | 0.127 |
| 90 | 0.446 | 0.401 | 0.355 | 0.394 | 0.345 | 0.382 | 0.338 | 0.297 | 0.335 | 0.290 |
| 100 | 1.000 | 1.000 | 1.000 | 1.000 | 1.000 | 1.000 | 1.000 | 1.000 | 1.000 | 1.000 |

Probabilities based on data published by Anderegg et al.[11]

**Supplementary Table 4 – Estimated annual cost for low/moderate and high intensity statins**

| **Statin intensity** | **Statin** | **Daily dose** | **Unit Cost, 2018 Baht**^a^ | **Proportion of low/moderate or high intensity statin use among Thai TAHOD sites^b^** | **Contribution to average unit cost, Baht** | **Average unit cost (sum of contributions), Baht** | **Average annual cost, Baht** |  |
| --- | --- | --- | --- | --- | --- | --- | --- | --- |
|  |  |  |  |  |  |  |  |  |
| Low/moderate | Atorvastatin | 10mg | 10.50 | 10.1% | 1.06 | 6.30 | 2,301 |  |
|  |  | 20mg | 15.15 | 19.2% | 2.91 |  |  |  |
|  | Fluvastatin | 80mg | 21.77 | 0.5% | 0.10 |  |  |  |
|  | Pitavastatin | 2mg | 22.40 | 4.1% | 0.91 |  |  |  |
|  | Pravastatin | 20mg | 20.72 | 0.3% | 0.05 |  |  |  |
|  |  | 40mg | 33.17 | 0.3% | 0.08 |  |  |  |
|  | Rosuvastatin | 5mg | 8.60 | 0.3% | 0.03 |  |  |  |
|  |  | 10mg | 17.20 | 4.3% | 0.74 |  |  |  |
|  | Simvastatin | 10mg | 0.50 | 24.6% | 0.12 |  |  |  |
|  |  | 20mg | 0.75 | 33.7% | 0.25 |  |  |  |
|  |  | 40mg | 1.35 | 2.7% | 0.04 |  |  |  |
| High | Atorvastatin | 40mg | 25.00 | 92.7% | 23.17 | 25.99 | 9,486 |  |
|  |  | 80mg | 50.00 | 4.4% | 2.19 |  |  |  |
|  | Rosuvastatin | 20mg | 21.40 | 2.9% | 0.63 |  |  |  |

Costs can be converted to $US by dividing by 31.16. ^a^ Unit costs based on those published by the Thai National Drug System Development Committee.[28] ^b^ Based on a survey of statin use among antiretroviral therapy users at Thai TAHOD sites contributing to our study population. TAHOD, TREAT Asia HIV Observational Database

**Supplementary Figure 2 – Tornado plot showing impact of changes in model parameters on the incremental cost-effectiveness ratio for statin initiation threshold ≥5% ASCVD risk versus ≥7.5% ASCVD risk**

**
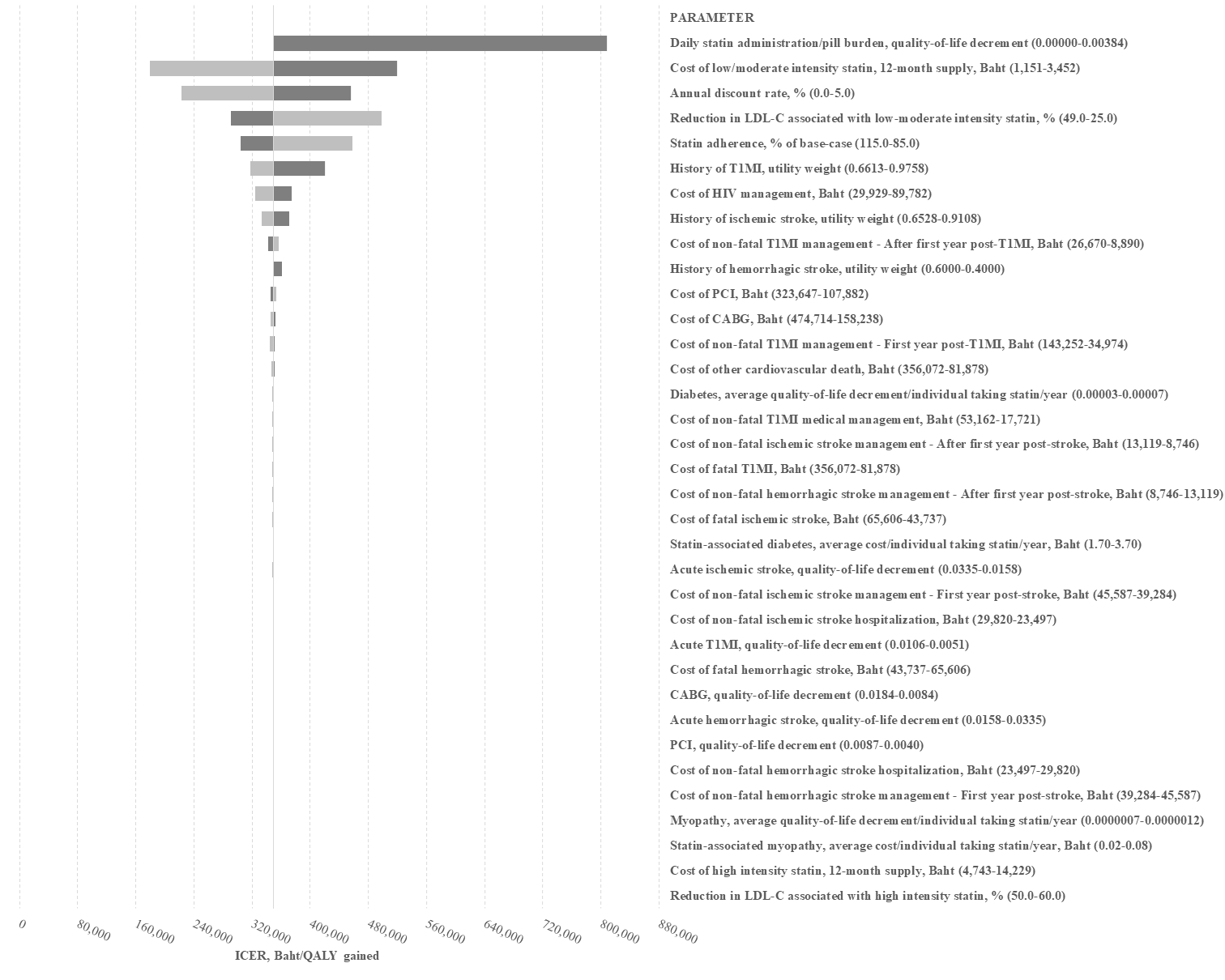
**

Shading of bars indicates directionality: lighter bars represent the smaller values in the sensitivity range and darker bars indicate the larger values. Directionality also indicated by the order of values shown in the text description. Cost-effectiveness based on a willingness-to-pay threshold of 160,000 Baht/QALY gained. Baht can be converted to $US by dividing by 31.16. ASCVD, cardiovascular disease; LDL-C, low density lipoprotein cholesterol; T1MI, type 1 myocardial infarction; CABG, coronary artery bypass graft; PCI, percutaneous coronary intervention; ICER, incremental cost-effectiveness ratio; QALY, quality-adjusted life-year

**Supplementary Material References**

1. Zhou J, Kumarasamy N, Ditangco R, Kamarulzaman A, Lee CK, Li PC, et al. **The TREAT Asia HIV Observational Database: baseline and retrospective data**. *Journal of acquired immune deficiency syndromes* 2005; 38(2):174-179.

2. Cholesterol Treatment Trialists Collaborators. **The effects of lowering LDL cholesterol with statin therapy in people at low risk of vascular disease: meta-analysis of individual data from 27 randomised trials**. *Lancet* 2012; 380(9841):581-590.

3. Dhillon S, Sabin CA, Alagaratnam J, Bagkeris E, Post FA, Boffito M, et al. **Level of agreement between frequently used cardiovascular risk calculators in people living with HIV**. *HIV medicine* 2019; 20(5):347-352.

4. Friis-Moller N, Ryom L, Smith C, Weber R, Reiss P, Dabis F, et al. **An updated prediction model of the global risk of cardiovascular disease in HIV-positive persons: The Data-collection on Adverse Effects of Anti-HIV Drugs (D:A:D) study**. *Eur J Prev Cardiol* 2016; 23(2):214-223.

5. Feinstein MJ, Hsue PY, Benjamin LA, Bloomfield GS, Currier JS, Freiberg MS, et al. **Characteristics, Prevention, and Management of Cardiovascular Disease in People Living With HIV: A Scientific Statement From the American Heart Association**. *Circulation* 2019:CIR0000000000000695.

6. Grundy SM, Stone NJ, Bailey AL, Beam C, Birtcher KK, Blumenthal RS, et al. **2018 AHA/ACC/AACVPR/AAPA/ABC/ACPM/ADA/AGS/APhA/ASPC/NLA/PCNA Guideline on the Management of Blood Cholesterol: A Report of the American College of Cardiology/American Heart Association Task Force on Clinical Practice Guidelines**. *J Am Coll Cardiol* 2019; 73(24):e285-e350.

7. Boettiger DC, Kerr S, Chattranukulchai P, Siwamogsatham S, Avihingsanon A. **Maintenance of statin therapy among people living with HIV**. *Aids* 2020; (in press).

8. Thiebaut R, El-Sadr WM, Friis-Moller N, Rickenbach M, Reiss P, Monforte AD, et al. **Predictors of hypertension and changes of blood pressure in HIV-infected patients**. *Antiviral therapy* 2005; 10(7):811-823.

9. Ledergerber B, Furrer H, Rickenbach M, Lehmann R, Elzi L, Hirschel B, et al. **Factors associated with the incidence of type 2 diabetes mellitus in HIV-infected participants in the Swiss HIV Cohort Study**. *Clin Infect Dis* 2007; 45(1):111-119.

10. Schauer GL, Malarcher AM, Asman KJ. **Trends in the Average Age of Quitting Among U.S. Adult Cigarette Smokers**. *Am J Prev Med* 2015; 49(6):939-944.

11. Anderegg N, Johnson LF, Zaniewski E, Althoff KN, Balestre E, Law M, et al. **All-cause mortality in HIV-positive adults starting combination antiretroviral therapy: correcting for loss to follow-up**. *Aids* 2017; 31 Suppl 1:S31-S40.

12. Benjamin EJ, Blaha MJ, Chiuve SE, Cushman M, Das SR, Deo R, et al. **Heart Disease and Stroke Statistics-2017 Update: A Report From the American Heart Association**. *Circulation* 2017; 135(10):e146-e603.

13. Cabral NL, Nagel V, Conforto AB, Amaral CH, Venancio VG, Safanelli J, et al. **Five-year survival, disability, and recurrence after first-ever stroke in a middle-income country: A population-based study in Joinvile, Brazil**. *Int J Stroke* 2018; 13(7):725-733.

14. Callaly E, Ni Chroinin D, Hannon N, Marnane M, Akijian L, Sheehan O, et al. **Rates, Predictors, and Outcomes of Early and Late Recurrence After Stroke: The North Dublin Population Stroke Study**. *Stroke; a journal of cerebral circulation* 2016; 47(1):244-246.

15. Chinwong D, Patumanond J, Chinwong S, Siriwattana K, Gunaparn S, Hall JJ, et al. **Clinical indicators for recurrent cardiovascular events in acute coronary syndrome patients treated with statins under routine practice in Thailand: an observational study**. *BMC Cardiovasc Disord* 2015; 15:55.

16. Edwards JD, Kapral MK, Fang J, Swartz RH. **Long-term morbidity and mortality in patients without early complications after stroke or transient ischemic attack**. *CMAJ* 2017; 189(29):E954-E961.

17. Lovett JK, Coull AJ, Rothwell PM. **Early risk of recurrence by subtype of ischemic stroke in population-based incidence studies**. *Neurology* 2004; 62(4):569-573.

18. Sabin CA, Ryom L, d'Arminio Monforte A, Hatleberg CI, Pradier C, El-Sadr W, et al. **Abacavir use and risk of recurrent myocardial infarction**. *Aids* 2018; 32(1):79-88.

19. Smolina K, Wright FL, Rayner M, Goldacre MJ. **Long-term survival and recurrence after acute myocardial infarction in England, 2004 to 2010**. *Circ Cardiovasc Qual Outcomes* 2012; 5(4):532-540.

20. Srimahachota S, Boonyaratavej S, Kanjanavanit R, Sritara P, Krittayaphong R, Kunjara-Na-ayudhya R, et al. **Thai Registry in Acute Coronary Syndrome (TRACS)--an extension of Thai Acute Coronary Syndrome registry (TACS) group: lower in-hospital but still high mortality at one-year**. *Journal of the Medical Association of Thailand = Chotmaihet thangphaet* 2012; 95(4):508-518.

21. Touze E, Varenne O, Chatellier G, Peyrard S, Rothwell PM, Mas JL. **Risk of myocardial infarction and vascular death after transient ischemic attack and ischemic stroke: a systematic review and meta-analysis**. *Stroke; a journal of cerebral circulation* 2005; 36(12):2748-2755.

22. Boettiger DC, Newall AT, Chattranukulchai P, Chaiwarith R, Khusuwan S, Avihingsanon A, et al. **Statins for atherosclerotic cardiovascular disease prevention in people living with HIV in Thailand: a cost-effectiveness analysis**. *Journal of the International AIDS Society* 2020; 23 Suppl 1:e25494.

23. Cao CF, Li SF, Chen H, Song JX. **Predictors and in-hospital prognosis of recurrent acute myocardial infarction**. *J Geriatr Cardiol* 2016; 13(10):836-839.

24. Gao M, Zheng Y, Zhang W, Cheng Y, Wang L, Qin L. **Non-high-density lipoprotein cholesterol predicts nonfatal recurrent myocardial infarction in patients with ST segment elevation myocardial infarction**. *Lipids Health Dis* 2017; 16(1):20.

25. Nakashima H, Mashimo Y, Kurobe M, Muto S, Furudono S, Maemura K. **Impact of Morning Onset on the Incidence of Recurrent Acute Coronary Syndrome and Progression of Coronary Atherosclerosis in Acute Myocardial Infarction**. *Circ J* 2017; 81(3):361-367.

26. Sattar N, Preiss D, Murray HM, Welsh P, Buckley BM, de Craen AJ, et al. **Statins and risk of incident diabetes: a collaborative meta-analysis of randomised statin trials**. *Lancet* 2010; 375(9716):735-742.

27. Stone NJ, Robinson JG, Lichtenstein AH, Bairey Merz CN, Blum CB, Eckel RH, et al. **2013 ACC/AHA guideline on the treatment of blood cholesterol to reduce atherosclerotic cardiovascular risk in adults: a report of the American College of Cardiology/American Heart Association Task Force on Practice Guidelines**. *J Am Coll Cardiol* 2014; 63(25 Pt B):2889-2934.

28. National Drug System Development Committee. **Royal Thai Government Gazette - National Drug Prices, 2018.** Available at: <http://www.dmsic.moph.go.th/dmsic/force_down.php?f_id=735> (accessed 1 Jul 2019)
